# Association of healthy lifestyle including a healthy sleep pattern with incident type 2 diabetes mellitus among individuals with hypertension

**DOI:** 10.1186/s12933-021-01434-z

**Published:** 2021-12-18

**Authors:** Zimin Song, Ruotong Yang, Wenxiu Wang, Ninghao Huang, Zhenhuang Zhuang, Yuting Han, Lu Qi, Ming Xu, Yi-da Tang, Tao Huang

**Affiliations:** 1 Department of Epidemiology and Biostatistics, School of Public Health, Peking University Health Science Center, 38 Xueyuan Road, Beijing, 100191 China; 2 Department of Epidemiology, School of Public Health and Tropical Medicine, Tulane University, New Orleans, LA USA; 3 Department of Nutrition, Harvard T.H. Chan School of Public Health, Boston, MA USA; 4 Channing Division of Network Medicine, Department of Medicine, Brigham and Women’s Hospital and Harvard Medical School, Boston, MA USA; 5 Department of Cardiology and Institute of Vascular Medicine, Peking University Third Hospital; Key Laboratory of Molecular Cardiovascular Sciences (Peking University), Ministry of Education; NHC Key Laboratory of Cardiovascular Molecular Biology and Regulatory Peptides; Key Laboratory of Cardiovascular Receptors Research, Beijing, 100191 China; 6 State Key Laboratory of Natural and Biomimetic Drugs, School of Pharmaceutical Sciences, Peking University, Beijing, 100191 China; 7 Center for Intelligent Public Health, Academy for Artificial Intelligence, Peking University, Beijing, China

**Keywords:** Hypertension, Type 2 diabetes mellitus, UK biobank, Lifestyle pattern

## Abstract

**Background:**

Evidence is limited regarding the association of healthy lifestyle including sleep pattern with the risk of complicated type 2 diabetes mellitus (T2DM) among patients with hypertension. We aimed to investigate the associations of an overall healthy lifestyle including a healthy sleep pattern with subsequent development of T2DM among participants with hypertension compared to normotension, and to estimate how much of that risk could be prevented.

**Methods:**

This study examined six lifestyle factors with T2DM cases among hypertension (227,966) and normotension (203,005) and their interaction in the UK Biobank. Low-risk lifestyle factors were defined as standard body mass index (BMI), drinking alcohol in moderation, nonsmoking, engaging in moderate- to vigorous-intensity physical activity, eating a high-quality diet, and maintaining a healthy sleep pattern.

**Results:**

There were 12,403 incident T2DM cases during an average of 8.63 years of follow-up. Compared to those with 0 low-risk lifestyle factors, HRs for those with 5–6 were 0.14 (95% CI 0.10 to 0.19) for hypertensive participants, 0.13 (95% CI 0.08 to 0.19) for normotensive participants, respectively (p_trend_ < 0.001). 76.93% of hypertensive participants were considerably less likely to develop T2DM if they adhered to five healthy lifestyle practices, increased to 81.14% if they followed 6-factors (with a healthy sleep pattern). Compared with hypertension adults, normotensive people gain more benefits if they stick to six healthy lifestyles [Population attributable risk (PAR%) 83.66%, 95% CI 79.45 to 87.00%, p for interaction = 0.0011].

**Conclusions:**

Adherence to a healthy lifestyle pattern including a healthy sleep pattern is associated with lower risk of T2DM in hypertensives, and this benefit is even further in normotensives.

**Supplementary Information:**

The online version contains supplementary material available at 10.1186/s12933-021-01434-z.

## Introduction

Type 2 diabetes mellitus (T2DM) has contributed tremendously to the burden of mortality and disability, with approximately a 425 million adults living with diabetes worldwide in 2017, estimated to rise to 642 million by 2040 [1]. People with elevated blood pressure confer excess risk of incident T2DM independent of BMI or other conventional diabetes risk factors [2–4]. Given the commonality in risk factors between T2DM and hypertension, it is of particular importance to identify effective strategies to prevent or delay the development of T2DM among patients with hypertension.

Strong evidence indicates that many cases of T2DM could be prevented by maintaining healthy body weight, following a high-quality diet, engaging in moderate to vigorous physical activity, avoiding smoking and consuming alcohol in moderation in general populations [1, 5, 6] and high-risk individuals such as raised fasting plasma glucose patients [7]. Individuals with elevated blood pressure also represent high-risk groups for the progression of T2DM [4]. However, whether the lifestyle prevention benefits on reducing T2DM events extend to hypertensive patients and whether these benefits differ between hypertensive and normotensive individuals are less established.

In addition to the common lifestyle factors, epidemiological studies have shown that sleep disturbances—comprising shorten time [8], difficulty in maintaining sleep [9], and mistimed sleep [10] increase the risk of T2DM. Meanwhile, a healthy sleep pattern as a lifestyle component was recently constructed and proven to be efficacious in T2DM prevention [11, 12]. Sleep is a natural fit for the sort of lifestyle, which may interact with other lifestyles to modify T2DM risk. For example, subjects reporting recurrent short sleep or late chronotypes have different meal patterns than those who sleep longer or morning chronotypes [13]. Later sleep timing has also been associated with a higher BMI, with late sleepers increasing snack intake and consuming more calories both at dinner and after [14]. The potential relevance of sleep pattern and these lifestyles may collaboratively prompt the T2DM incident. However, no prospective studies regarding the association of a healthy lifestyle including a healthy sleep pattern with T2DM has been reported, and as of yet, it remains unclear whether such associations are different among hypertensive and normotensive individuals.

Therefore, to fill these critical knowledge gaps, we aimed to prospectively investigate the associations of healthy lifestyle including a healthy sleep pattern with incident T2DM in individuals with hypertension compared with normotension within UK Biobank.

## Methods

### Study population

The UK Biobank cohort study includes more than 500,000 participants (aged 40–70 years) recruited from 22 assessment centres between 2006 and 2010 from the general population (5.5% response rate) across the United Kingdom [15]. Participants provided information on lifestyle and other potentially health-related aspects through extensive baseline questionnaires, interviews, and physical measurements. All participants provided written informed consent for the study [16]. The UK Biobank study has approval from the North West Multi-center Research Ethics Committee [17]. More information about study design and population have been described in detail previously [18, 19].

In the present analysis, we excluded participants who reported previous medical histories of cancer (n = 35,433); had prevalent diabetes (n = 36,100) defined if either: (1) fasting plasma glucose (FPG) ≥ 7 mmol/L or haemoglobin A_1c_(HbA_1c_) ≥ 6.5% on a peripheral blood test at baseline. (2) Self-reported “diabetes diagnosed by a doctor” plus self-report of currently taking antidiabetic medication. After these exclusions, a total of 430,971 participants remained for the current analysis (Additional file 1: Fig. S1). When we modelled lifestyle factors to T2DM diagnosis, participants with missing information of lifestyle factors including body mass index (BMI), smoking status, alcohol intake, physical activity, dietary data or sleep pattern were further excluded. These exclusion criteria were based on minimizing reverse causation bias and reducing the impact of measurement errors and missing data. After exclusions, 12,403 incident T2D cases were included in the final analysis, with an average of 8.63 years of follow-up.

### Definition of a healthy lifestyle score

A healthy lifestyle score was constructed based on six modifiable lifestyle factors—BMI, smoking status, alcohol consumption, physical activity, diet and sleep pattern, according to previous studies or The American Diabetes Association (ADA) recommendations for diabetes care [11, 20, 21]. Participants scored 1 point if he or she was classified as low risk for that factor or 0 points otherwise for each of 6 lifestyle factors. The sum of the six factors constituted a final healthy lifestyle score of 0, 1, 2, 3, 4 or ≥ 5 (higher score for a healthier lifestyle). Details of the assessments of individual lifestyle factors can be found in Additional file 1: Table S2.

### BMI, cigarette smoking

For general adiposity measured by BMI, the low-risk was defined as those who had a BMI of ≤ 24.9 kg/m^2^ for that overweight and obesity are associated with increased risk of T2DM [22]. For smoking, we described low-risk as nonsmokers.

### Alcohol consumption

For alcohol consumption, the low-risk was categorized as moderate alcohol consumption: 0 to 15 g/day for women and 0 to 28 g/day for men, with moderate consumption coordinated with a lower risk of T2DM [23]. Of note, few participants drank heavily in our cohorts; < 1% of total participants drank alcohol > 90 g/day.

### Physical activity

We classified those who meet the American Heart Association recommendations of regular physical activity as low risk: ≥ 150 min of moderate physical activity; or ≥ 75 min of vigorous physical exercise; or ≥ 150 min of combined moderate and vigorous physical activity; or moderate physical activity at least 5 days a week; or vigorous activity once a week [24].

### Dietary intake

We characterized low-risk as more than four points of a healthy diet score that contained five low-risk diet factors including fruit ≥ 3 servings/day, vegetable ≥ 3 servings/day, (shell)fish ≥ 2 servings/week, processed meats ≤ 1 serving/week, unprocessed meats ≤ 2 serving/week, which was based on recommendations on dietary priorities for cardiometabolic health and improvement of a range of risk factors linked to better glucose control and reduced T2DM risk [25] (Additional file 1: Table S3).

### Sleep patterns

Five low-risk sleep factors (early chronotype; sleep 7–8 h per day; reported never or rarely insomnia symptoms; no self-reported snoring; and no frequent daytime sleepiness) were included to generate a healthy sleep score ranging from 0 to 5 (one point was given for each low-risk sleep factor), which was associated with a lower risk of incident T2D [11] (Additional file 1: Table S4). We then define a healthy sleep pattern as healthy sleep score ≥ 4 points.

### Assessment of covariates and hypertension

Covariate information was inquired by baseline questionnaire including sociodemographic characteristics, personal and family medical history. Socioeconomic status is provided by quintiles of the Townsend deprivation index, combining information on social class, employment, car availability, and housing [26]. A participant was considered a family history of T2DM or hypertension if he or she reported at least one first-degree relative with that disease. Prevalent hypertension was defined if either: (1) medical records from the National Health Service (NHS) Information Centre for participants from England and Wales and the NHS Central Register Scotland for participants from Scotland (2) mean BP at baseline was greater than clinically defined criteria over two measurements (systolic BP ≥ 140 mmHg or diastolic BP ≥ 90 mmHg). (3) Self-reported “hypertension diagnosed by a doctor” plus self-report of currently taking antihypertensive medication.

### Ascertainment of T2DM

T2DM was ascertained using hospital inpatient records containing data on admissions and diagnoses obtained from the Hospital Episode Statistics (HES) for England, Scottish Morbidity Record data for Scotland (SMR) for Scotland, and the Patient Episode Database for Wales (PEDW). Diagnoses were recorded using the International Classification of Diseases (ICD), Ninth and Tenth Revision coding system (Additional file 1: Table S1).

### Statistical analysis

Baseline characteristics of all participants were summarized as means with standard deviation for continuous variables and percentages for categorical variables. Person-time for each participant was calculated from the date of enrollment to T2DM diagnosis, death, or the end of follow-up (HES Mar 31, 2017; SMR Oct 31, 2016; PEDW Feb 29, 2016.), whichever came first. Poisson regression models were used to derive adjusted incidence rates of T2DM. Cox proportional hazards models were applied to estimate hazard ratios (HRs) and 95% confidence intervals (CIs) for the associations of each healthy lifestyle factor and the combined healthy lifestyle score with the risk of T2DM events among participants with or without hypertension. Proportional hazards assumption was tested using Schoenfeld residuals. In multivariate models, we adjusted for age, sex, educational attainment, socioeconomic status, family history of diabetes or hypertension. In analyzing the association between each component of the healthy lifestyle score and T2DM events, we further adjusted for all other parts. Analyses were further stratified by age, sex, educational attainment, socioeconomic status, family history of diabetes or hypertension and healthy lifestyle factors. The linear trend was performed by treating the healthy lifestyle score as a continuous variable. To test whether the pattern of association varies across stratifications, we estimated multiplicative interactions by including the product term (exposure × stratification variable) in the models using the likelihood ratio test. Several sensitivity analyses were conducted to demonstrate the robustness of our findings. Individual low-risk lifestyle factors and healthy lifestyle scores were evaluated to estimate the proportion of the population that might be attributable to unhealthy lifestyle patterns (population-attributable risk [PAR]%). The PAR% was calculated using the formula p(HR − 1)/(1 + p[HR − 1]), where p is the prevalence of individuals not in the low-risk group and HR is the associated multivariable-adjusted hazard ratio of those individuals. 2-sided p < 0.05 was considered statistically significant. All statistical analyses were performed using Stata (version 13.1, StataCorp, College Station, Texas).

## Results

### Baseline characteristics of included participants

The characteristics of the study participants with or without hypertension are shown in Table 1. The mean age of all participants was 56.04 ± 8.13 years. Of 430,971 participants, the proportions of participants with 3, 4, and ≥ 5 healthy lifestyle factors were 22.37%, 14.33% and 5.77% in overall and 27.25%, 14.96% and 5.12% in hypertension, respectively. Compared with participants with normotension, participants with hypertension were older, had higher proportions of men, were less educated, were more likely to have a family history of hypertension, and had a smaller number of more than three healthy lifestyle factors.

**Table 1:** Baseline characteristics of participants with hypertension and incident T2DM in UK Biobank

Baseline characteristic	Hypertension		Normotension	
	No. of hypertension (n = 227,966)	Incident T2DM (n = 5428)	No. of normotension (n = 203,005)	Incident T2DM (n = 1547)
Age, mean (SD), y	58.33 (7.52)	59.67 (7.13)	53.47 (8.02)	56.64 (7.97)
Male	117,456 (51.52)	3210 (59.14)	78,392 (38.62)	808 (52.23)
Education				
Vocational	16,505 (7.39)	472 (9.07)	11,155 (5.60)	127 (8.67)
Lower secondary	59,766 (26.75)	1324 (25.45)	54,733 (27.5)	373 (25.48)
Upper secondary	23,628 (10.57)	405 (7.78)	24,629 (12.37)	140 (9.56)
Higher	77,190 (34.54)	1245 (23.93)	85,783 (43.10)	432 (29.51)
None of the above	46,364 (20.75)	1757 (33.77)	22,724 (11.42)	392 (26.78)
Family history of T2DM	40,909 (17.95)	1552 (28.59)	34,640 (17.06)	476 (30.77)
Family history of hypertension	95,551 (41.91)	2057 (37.90)	68,326 (33.66)	376 (24.31)
Socioeconomic status quintile				
1 (least deprived)	46,326 (20.35)	760 (14.02)	41,336 (20.39)	218 (14.10)
2–4	137,380 (60.33)	3039 (56.05)	122,007 (60.18)	821 (53.10)
5 (most deprived)	43,991 (19.32)	1623 (29.93)	39,407 (19.44)	507 (32.79)
Systolic blood pressure, mean (SD), mmHg	149.59 (15.85)	148.55 (17.78)	123.69 (9.89)	126.10 (9.23)
Diastolic blood pressure, mean (SD), mmHg	87.71 (9.35)	87.09 (10.34)	76.14 (7.00)	77.66 (6.94)
No. of healthy lifestyle score				
0	8105 (4.51)	393 (10.03)	4462 (2.73)	87 (7.87)
1	33,067 (18.40)	1127 (28.77)	20,266 (12.41)	288 (26.04)
2	53,509 (29.77)	1283 (32.75)	40,597 (24.87)	355 (32.10)
3	48,976 (27.25)	792 (20.22)	47,421 (29.05)	260 (23.51)
4	26,895 (14.96)	273 (6.97)	34,859 (21.35)	89 (8.05)
≥ 5	9203 (5.12)	49 (1.25)	15,655 (9.59)	27 (2.44)
Incident diabetes per 1000 person-years^a^	2.42 (95% CI 2.35–2.49)		1.01 (95% CI 0.95–1.06)	

Data are mean (SD) or percentages, or as otherwise indicated; Percentages may not sum to 100 because of rounding

*T2DM* Type 2 Diabetes Mellitus, *BMI* Body Mass Index, *No*. number, *CI* confidence interval

^a^Adjusted for age, sex, education, socioeconomic status quintile, family history of T2DM and hypertension

### Incidence of type 2 diabetes mellitus

A total of 6975 incident T2DM cases were documented during a mean follow-up of 8.63 years (incidence rate 1.87 per 1000 person-year). In contrast, the multivariable-adjusted incidence rate of T2DM events was higher in participants with hypertension relative to those without [2.42 (95% CI 2.35–2.49) vs 1.01 (95% CI 0.95–1.06) per 1000 person-years; p < 0.001] (Table 1).

### Associations of individual healthy lifestyle factors with incident T2DM

When the six lifestyle factors were collapsed into binary categories of low risk vs high risk (reference group), with the exception of a healthy diet, all other five factors (ideal BMI, moderate alcohol consumption, nonsmoking, physical activity at goal, healthy sleep score) were each independently associated with a 70.80%, 18.25%, 23.00%, 29.79%, 25.51% lower risk of incident T2DM in participants affected by hypertension, respectively (Table 2). Similar associations but more significant in magnitude were observed in normotension and overall participants for the five healthy lifestyle factors mentioned above. Different from participants with hypertension, a healthy diet was associated with decreased risks of T2DM among normotension and overall. Consistent results were observed when analyses were further stratified into subgroups by individual lifestyle factor (Additional file 1: Table S6). There were no significant differences by blood pressure status (all p for interaction > 0.05) in the association of baseline individual healthy lifestyle factor with risk of T2DM.

**Table 2:** Multivariable-adjusted HRs (95% CIs) and PAR% (95% CIs) for T2DM events by low-risk lifestyle factors

Category	No. of participants^a^	Cases/PYs (1000)^b^	HR (95% CI)^c^	Risk difference (%)	PAR% (95%CI)
Hypertension					
BMI	56,608	0.83	0.29 (0.26–0.33)	70.80	65.32 (61.96–68.38)
Alcohol consumption	108,626	2.36	0.82 (0.77–0.87)	18.25	10.71 (7.52–13.79)
Smoking status	121,704	2.23	0.77 (0.72–0.82)	23.00	13.40 (10.38–16.31)
Physical activity	156,717	2.30	0.70 (0.66–0.75)	29.79	11.42 (9.62–13.18)
Diet score	27,772	2.33	0.96 (0.86–1.07)	4.11	3.68 (-5.82–12.33)
Sleep pattern	65,552	1.93	0.74 (0.69–0.80)	25.51	18.81 (14.72–22.70)
Per unit increase in healthy lifestyle score	NA	NA	0.70 (0.68–0.72)	29.67	NA
Normotension					
BMI	90,976	0.28	0.26 (0.22–0.31)	73.97	63.33 (59.29–66.96)
Alcohol consumption	103,622	0.74	0.80 (0.71–0.90)	19.97	11.06 (5.45–16.34)
Smoking status	116,793	0.65	0.80 (0.70–0.90)	20.32	11.25 (5.63–16.55)
Physical activity	146,193	0.71	0.74 (0.65–0.84)	26.28	9.33 (5.94–12.60)
Diet score	26,502	0.58	0.72 (0.58–0.89)	28.09	25.61 (10.27–38.33)
Sleep pattern	68,672	0.57	0.72 (0.63–0.83)	27.57	19.76 (12.47–26.45)
Per unit increase in healthy lifestyle score	NA	NA	0.65 (0.62–0.68)	35.08	NA
All participants					
BMI	147,584	0.49	0.25 (0.23–0.28)	74.90	68.00 (65.70–70.14)
Alcohol consumption	212,249	1.57	0.79 (0.75–0.84)	20.61	11.94 (9.26–14.54)
Smoking status	238,497	1.45	0.78 (0.74–0.83)	21.59	12.44 (9.78–15.02)
Physical activity	302,910	1.53	0.70 (0.66–0.75)	29.61	11.16 (9.60–12.70)
Diet score	54,274	1.47	0.90 (0.82–0.99)	9.66	8.69 (0.71–16.03)
Sleep pattern	134,224	1.23	0.73 (0.69–0.78)	26.90	19.71 (16.23–23.05)
Per unit increase in healthy lifestyle score	NA	NA	0.67 (0.66–0.69)	32.86	NA

*T2DM* Type 2 Diabetes Mellitus, *HR* hazard ratio, *CI* confidence interval, *PAR*% population-attributable risk percent

^a^Participants in the low-risk group

^b^Incidence density per 1000 person-years

^c^Low-risk lifestyle factors: BMI of ≤ 24.9 kg/m^2^; moderate alcohol consumption (0 to 15 g/day for women and 0 to 28 g/day for men). Nonsmoking; moderate to vigorous physical activity ($150 min/week), high-quality diet (top two-fifths of healthy diet score), and healthy sleep pattern (top two-fifths of healthy diet score). Adjusted for age, sex, education, socioeconomic status, parental history of T2DM or hypertension

### Association of a healthy sleep pattern with incident T2DM

**Fig. 1:**
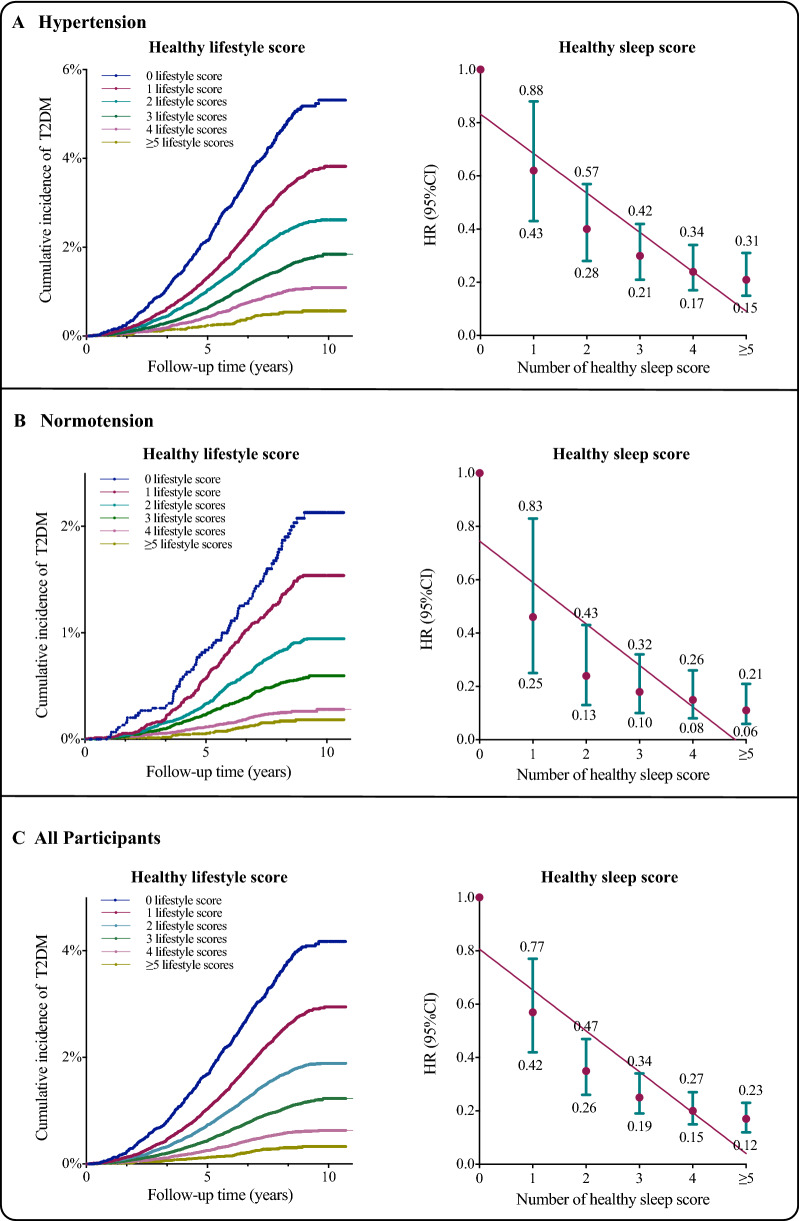
Effect of lifestyle factors on the risk of incident T2DM among overall and hypertension population in the UKB cohort. HRs for each group were compared with those with no healthy lifestyle factors; error bars show 95% CIs. Low-risk lifestyle factors: BMI of ≤ 24.9 kg/m^2^; moderate alcohol consumption (0 to 15 g/day for women and 0 to 28 g/day for men). Nonsmoking; moderate to vigorous physical activity ($150 min/week), high-quality diet (top two-fifths of healthy diet score), and healthy sleep pattern (top two-fifths of healthy sleep score). Low-risk sleep factors: early chronotype; sleep 7–8 h per day; reported never or rarely insomnia symptoms; no self-reported snoring; and no frequent daytime sleepiness. Both models were adjusted for age, sex, education, socioeconomic status, parental history of T2DM or hypertension. *T2DM* Type 2 Diabetes Mellitus, *HR* hazard ratio; *CI* confidence interval. All p for trend < 0.0001, calculated using the log-rank test

Regarding T2DM events, participants with the highest healthy sleep score in comparison with the lowest score showed 79% (HR 0.21, 95% CI 0.15 to 0.31), 89% (HR 0.11, 95% CI 0.06 to 0.21), and 83% (HR 0.17, 95% CI 0.12 to 0.23) lower risk of T2DM in hypertension, normotension and all participants (Fig. 1 and Additional file 1: Table S7). After incremental adjustment for baseline hypercholesterolemia and the use of antihypertensive or cholesterol-lowering drugs, strong inverse associations were observed between a healthy sleep score as a continuous variable and T2DM outcome among hypertensive participants (HR 0.78, 95% CI 0.75 to 0.81, p trend < 0.001), which had been modified by blood status at baseline (p for interaction = 0.0334).

### Association of a healthy lifestyle pattern with incident T2DM

Compared to those with a healthy lifestyle score of 0, the fully adjusted HRs (95% CI) of those with a healthy lifestyle score of 5–6 was 0.14 (95% CI 0.10 to 0.19) among hypertension patients, 0.13 (95% CI 0.08 to 0.19) in normotension individuals and 0.12 (95% CI 0.09 to 0.15, all p trend < 0.001) among all participants, respectively (Fig. 1 and Additional file 1: Table S5). When evaluated ordinally, participants having 1-number increment in healthy lifestyle score was associated with a greater magnitude of T2DM risk-lowing in normotension (HR 0.65, 95% CI 0.62 to 0.68) than in hypertension (HR 0.70, 95% CI 0.68 to 0.72), indicating significant modification effects of blood pressure status on the associations (p for interaction = 0.0011) (Table 2).

Concordant results were obtained when analyses were stratified by age at baseline, sex, education levels, socioeconomic status, and family history of hypertension or T2DM (Table 3). For normotensive participants, the inverse association between per unit increase in healthy lifestyle score and T2DM was particularly pronounced in females (p for interaction = 0.0042). For participants with hypertension, there was further evidence of a somewhat stronger association among the adults younger than 55 years (p for interaction < 0.0001) and without a family history of T2DM (p for interaction = 0.0086).

**Table 3:** Stratified analysis of the association of T2DM incidence with per 1-number increment in low-risk lifestyle factors

Categories	Cases	Hypertension		Normotension	
		HR (95% CI)^a^	p for interaction^b^	HR^a^ (95% CI)	p for interaction
Age, year			< 0.0001		0.0542
≤ 55	2061	0.66(0.62–0.69)		0.64(0.59–0.69)	
56–65	2980	0.69(0.67–0.72)		0.63(0.58–0.68)	
≥ 65	1934	0.76(0.72–0.80)		0.71(0.64–0.79)	
p_trend_ value		< 0.0001		< 0.0001	
Sex			0.3929		0.0042
Female	2957	0.71(0.68–0.75)		0.60(0.55–0.65)	
Male	4018	0.70(0.67–0.72)		0.69(0.64–0.74)	
Education			0.5461		0.6715
Vocational	599	0.65(0.59–0.71)		0.64(0.54–0.77)	
Lower secondary	1697	0.68(0.65–0.72)		0.64(0.58–0.71)	
Upper secondary	545	0.78(0.71–0.86)		0.63(0.54–0.74)	
Higher	1677	0.70(0.67–0.74)		0.66(0.60–0.71)	
None of the above	2149	0.71(0.68–0.75)		0.66(0.59–0.73)	
p_trend_ value		< 0.0001		< 0.0001	
Socioeconomic status			0.5976		0.0935
1 (least deprived)	978	0.69(0.64–0.73)		0.70(0.62–0.79)	
2–4	3860	0.72(0.69–0.74)		0.65(0.61–0.69)	
5 (most deprived)	2130	0.68(0.65–0.72)		0.63(0.57–0.69)	
p_trend_ value		< 0.0001		< 0.0001	
Family history of T2DM			0.0086		0.1108
No	4947	0.69(0.67–0.71)		0.63(0.59–0.67)	
Yes	2028	0.74 (0.71–0.78)		0.69(0.63–0.75)	
Family history of hypertension			0.4169		0.7651
No	4542	0.70 (0.67–0.72)		0.65(0.61–0.69)	
Yes	2433	0.71 (0.68–0.75)		0.64 (0.58–0.71)	

*T2DM* Type 2 Diabetes Mellitus, *HR* hazard ratio, *CI* confidence interval

Low-risk lifestyle factors: BMI of ≤ 24.9 kg/m^2^; moderate alcohol consumption (0 to 15 g/day for women and 0 to 28 g/day for men). nonsmoking; moderate to vigorous physical activity ($150 min/week), high-quality diet (top two-fifths of healthy diet score), and healthy sleep pattern (top two-fifths of healthy diet score)

^a^Hazard ratios (95% CI) were adjusted for age, sex, education, socioeconomic status quintile, family history of T2DM and hypertension

^b^p for interaction calculated using multiplicative interaction terms and application of the likelihood ratio test

### Sensitivity analysis

To test the robustness of the findings, we performed sensitivity analyses that included: (1) further adjustment for baseline hypercholesterolemia and the use of antihypertensive or cholesterol-lowering drugs in the primary analysis (Additional file 1: Tables S5, S7); (2) further adjustment for waist circumference (Additional file 1: Table S5); (3) examining HRs for T2DM among participants with dysglycaemia (Impaired fasting glucose [IFG] only; IFG and hypertension combined) at baseline (Additional file 1: Table S8), as it may be the pathway from risk to diabetes; (4) excluding participants whose T2DM outcomes occurred in the first 2 years of follow-up to minimize potential bias resulting from subclinical conditions (Additional file 1: Table S9); (5) excluding underweight participants (BMI < 18.5 kg/m^2^) in Additional file 1: Table S10; and (6) excluding individuals who reported sleep apnea at baseline in Additional file 1: Table S11. These sensitivity analyses did not materially alter the risk estimates.

### The population-attributable risk of T2DM

**Fig. 2:**
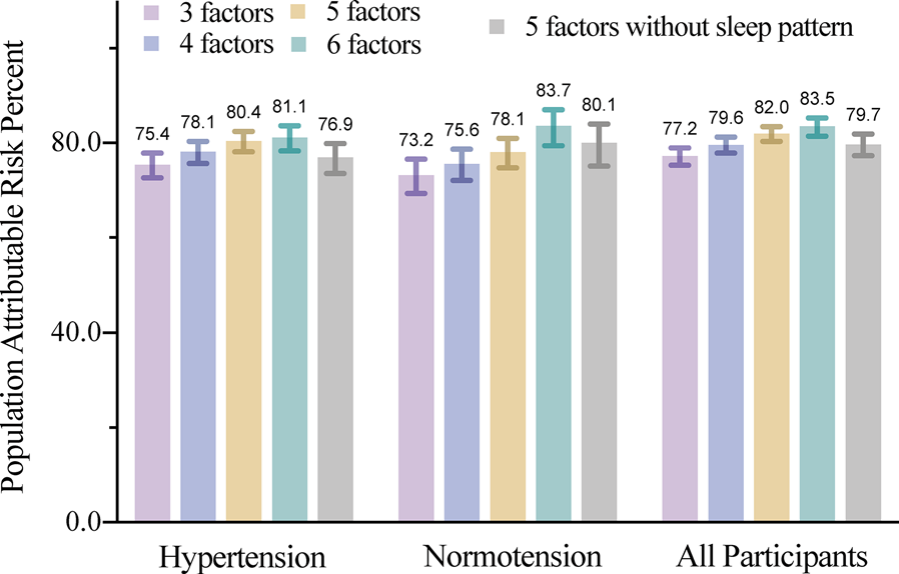
Multivariable-adjusted population-attributable risk percents (95% CI) for incident T2DM by combining low-risk lifestyle factors among 430,971 participants. The multivariable model was adjusted for age, sex, education, socioeconomic status quintile, family history of T2DM and hypertension. All six lifestyle factors were included simultaneously in the same model. The specific combination of low-risk lifestyle factors are as follows: three lifestyle factors indicating body mass index (BMI), healthy sleep pattern, smoking. The model was also adjusted for physical activity, healthy diet, and alcohol consumption; 4 factors included the first 3 factors and physical activity. The model was additionally adjusted for healthy diet and alcohol consumption; 5 factors including the first 4 factors and alcohol consumption. The model was additionally adjusted for healthy diet; 6 factors including the first 5 factors and healthy diet

Table 2 presents the PAR% for each healthy lifestyle factor. Compared with the low-risk group, each lifestyle factor was estimated to explain 8.69% (diet score 4–5) to 68.00% (BMI ≤ 25 kg/m^2^) of the population risk of developing T2DM among participants with hypertension. The combined PAR% of T2DM concerning BMI, poor sleep pattern, smoking was 75.43% (95% CI 72.69% to 77.90%), which increased to 78.13% (95% CI 75.69% to 80.34%) when additionally considering physical activity among hypertensive subjects (Fig. 2 and Additional file 1: Table S12). The PAR% of the lifestyle score of 5-factors (without the sleep pattern) was 76.93% (95% CI 73.56 to 79.87%) vs that of 6-factors (with a sleep pattern) was 81.14% (95% CI 78.29 to 83.61%) for participants with hypertension, suggesting that approximately four-fifths of T2DM events in this cohort would not have occurred if all participants had been in the low-risk group for all six lifestyle factors. The risk attributable to six modifiable lifestyle factors was larger in normotension (PAR% 83.66%, 95% CI 79.45 to 87.00%, p for interaction = 0.0011).

## Discussion

Based on this large, prospective cohort of middle-aged to older men and women, we examined the associations of the newly defined healthy lifestyle score integrating BMI, smoking status, alcohol consumption, physical activity, diet and sleep pattern with incident T2DM. When patients with hypertension have 5 or more compared with 0 ideal lifestyle factors, they have an 86% reduced risk of developing T2DM, which is slightly lower than normotensives. If these associations are causal, our results suggest that nearly 81% of T2DM events will not have occurred if all participants have been in the low-risk group for all six lifestyle factors, even with baseline hypertension.

### Comparison with other studies

Prehypertension, as well as hypertension, were strongly and independently associated with type 2 diabetes, even after controlling for potential confounders [3, 4, 27]. In Framingham Heart Study, the incident rates of T2DM in normotensive white women was 2.8 per 1000 person-years, while that of hypertensive white women was nearly 10.0 per 1000 person-years [3]. Our study also reported higher incident rates of T2DM in the hypertension group, which means what interventions to prevent or delay the onset of T2DM could be introduced in people with hypertension is essential.

T2DM is, in addition to genetic predisposition, largely dependent on behavioural factors such as dietary habits and physical activity. It is well established that a healthy lifestyle (BMI ≤ 24.9 kg/m^2^, nonsmoking, improving diet quality, promoting moderate-intensity physical activity, drinking alcohol in moderation) is associated with a lower risk of T2DM in largely healthy populations [28, 29], which is consistent with our current research results. Furthermore, sleep disturbance, characterizing the 24 h lifestyle of modern societies, has increasingly been shown to represent an additional lifestyle factor adversely affecting T2DM [8]. Our studies also found that getting proper sleep helps reduce the risk of T2DM. Existing evidence from different studies [11, 30, 31], together with our analysis, support the selection of healthy sleep pattern included in the healthy lifestyle pattern. In general, although many prospective studies including RCTs had confirmed the importance of lifestyle intervention in diabetes control, they might have missed sleep pattern which is an important lifestyle factor, or patients who already have hypertension. So that, in the current study, we innovatively integrated an overall healthy lifestyle pattern by taking into account the combined impact of five known lifestyle factors and a newly constructed healthy sleep pattern on T2DM risk. This new lifestyle pattern reflects a more comprehensive healthy lifestyle pattern which has the potential to delay if not prevent progression to type 2 diabetes in all populations, whether the distribution of blood pressure. Our findings added valuable information to the field of lifestyle modification in preventing diabetes among high-risk people.

It is worth mentioning that we observed there was a statistically significant interaction between healthy lifestyle score and blood pressure status after adjusting the covariates. Adopting this new lifestyle pattern could significantly reduce the risk of diabetes in people with hypertension, even if the effect seems to be weaker than that of the normotensive population. Based on our results, we urged that people with hypertension, particularly less than 55 years adopt a new lifestyle pattern, and in a more helpful recommendation, those normotensive people, especially females should follow it too-to prevent T2DM. In addition to lifestyle interventions, blood pressure management with antihypertensive drugs may be effective in reducing T2DM risk. It is worth mentioning that different antihypertensive drugs in T2DM prevention have yielded mixed results. Different from lifestyle intervention, some antihypertensive drugs may influence blood glucose metabolism while decreasing blood pressure. Diabetes Prevention Program excluded individuals who used thiazide diuretics or beta-blockers for the treatment of hypertension based on epidemiological studies and clinical trials suggesting a causal connection between beta-blockers and the development of T2DM [32]. A multi-center study showed that angiotensin-receptor blockers (ARBs) are effective at preventing the development of T2DM besides their antihypertensive effects [33]. Therefore, it is prudent to choose antihypertensive drugs to prevent diabetes and the adverse effects of drugs on blood glucose must be weighed against the proven benefits of lowering blood pressure and reducing the risk of cardiovascular events [34].

The previous study indicated that a total of 91% (95% CI 86 to 95%) of cases of type 2 diabetes could be attributed to the five unhealthy lifestyles (BMI, diet, physical activity, smoking and alcohol consumption) in Nurses’ Health Study (NHS) [29], 81% (95% CI 63 to 91%) in Health Professionals Follow-up Study (HPFS) [28], 89% (95% CI 23 to 99%) in the Cardiovascular Health Study in US [35], 78.8% (95% CI 67.9% to 86.3%) in the China Kadoorie Biobank [36], 86% (95% CI 64% to 95%) among Caucasians men and 70% (95% CI 44% to 85%) among Japanese Americans in the Multiethnic Cohort study [37]. In our new analyses, the population attributable risk for these five unhealthy lifestyle factors was 79.72% in the general population in the UK biobank, which was close to that of HPFS. More importantly, when the sleep pattern was incorporated into lifestyle pattern, PAR% increased to 83.51%, enhanced the protective effect of this new lifestyle pattern on diabetes.

Similarly, the PAR% results of all six unhealthy lifestyle factors were 81.14% among hypertensive people, lower than that in normotensive people. This may illustrate that only lifestyle intervention might not be enough for T2DM prevention in hypertension patients, although the efficacy of this new multicomponent lifestyle pattern had been confirmed.

### Potential mechanisms

Irregular sleep patterns were found to increase overall brain activation to food stimuli, which may have been associated with adverse metabolic traits such as obesity and disturbed glucose metabolism control [38, 39]. In accordance with these observations, findings from experimental studies have emphasized some distinct mechanisms by which sleep restriction might provoke decreases in glucose tolerance and insulin sensitivity [40]. In addition to their harmful effect in diabetes mellitus, a great piece of research published has suggested that insulin resistance and compensatory hyperinsulinemia mediate the reciprocal interaction between hypertension and T2DM [41, 42]. One common underlying mechanism is that insulin resistance had an effect on vascular endothelial dysfunction, which are closely linked to accelerated vascular stiffness, contributing to the progression of cardiovascular diseases such as hypertension [43, 44]. Additionally, hyperinsulinemia stimulated noradrenalin release, also contributing to elevating BP and simultaneously had an adverse influence on glucose metabolism [45]. This may be the potentially explanatory mechanism for why poor lifestyle habits may increase the risk of developing T2DM pathologies, especially among hypertension patients.

### Public health impact

The new healthy lifestyle pattern containing six modifiable lifestyle factors defined by this study provides a positive frame of reference for T2DM primary prevention. It is also of value in identifying high-risk individuals and facilitating health management. However, there are only 7.25% of participants adopted more than five healthy lifestyle factors in our study. Finding that a normal BMI, a healthy sleep pattern, and no smoking are three significant factors to prevent diabetes, lowering the risk of the disease by 75%. From a public health perspective, this epidemiological conclusion makes it easier for individuals to understand the importance of T2DM prevention and translate it into practice, therefore being more informative to the general population.

### Strengths and limitations

The strengths of this study included its population-based prospective study design, the large sample size of incident T2DM and the comprehensive analyses including individuals with hypertension, normotension. To the best of our knowledge, this is the first prospective study to comprehensively investigate the association of an overall healthy lifestyle pattern that integrate several healthy lifestyle factors and a healthy sleep pattern with incident T2DM risk.

The present study also has notable limitations. First, participants who were excluded owing to missing data on lifestyle factors included a slightly higher proportion of men. The results should be interpreted with caution because of the potential risk of selection bias. Second, the lifestyle factors used to create a healthy lifestyle score were evaluated only at baseline, so changes in the factors over time are not available in this study. Future cohort studies with repeated measurements of lifestyle factors would be required to confirm our findings and investigate the impact of dynamic changes in lifestyle patterns on T2DM outcomes. Third, measurement errors in self-reported assessments of lifestyle data were inevitable. However, this type of misclassification of exposure is likely to be non-differential related to the T2DM and thus would be more likely to bias the effect estimations toward the null. Fourth, our study did not have direct measurements of blood pressure control and the severity of hypertension. However, the results remained similar when we further adjusted for the use of antihypertensive medications, suggesting that our findings are unlikely to be explained by confounding due to the severity of hypertension. Fifth, interpreting the population attributable risk estimates assumes a causal association between the low-risk lifestyle factors and T2DM risk. This is a big assumption, given that our findings were based on an observational study. In addition, we could not entirely rule out confounding by genetic susceptibility, medication use, or psychosocial stress; residual confounding due to unknown or unmeasured factors remains possible. Finally, The UK Biobank had a low response rate in baseline survey: of the more than 9 million people who received an invitation to take part in, only around 6% responded [46]. Although the UK biobank aimed to be representative of the general population, it might be unrepresentative due to the ‘healthy volunteers’ selection bias. In fact, previous studies had been carried out to compare risk factor (demographic, social, behavioural, and biomedical)-endpoint associations in UK biobank with those from representative studies with conventional response rates, suggesting that risk factor associations in the UK Biobank seem to be generalisable [47]. Moreover, the study’s large sample size and a sufficient variance in the exposures of interest ensure the generalisability of the findings regarding risk factors and health outcomes [48]. We acknowledged that the present study was mainly based on European descent. Generalization of the results to other populations should be done with caution.

## Conclusions

Our study based on a large cohort indicates that adherence to a healthy lifestyle pattern including a healthy sleep pattern is associated with substantially lower risk of incident T2DM in hypertensive participants. Importantly, more cases of diabetes can be preventable by adherence to this combined lifestyle pattern in normotensives.

## Supplementary Information

**Additional file 1: Table S1.** Disease codes for Metabolic diseases of Type 2 Diabetes, Hypertension, Cardiovascular disease in UK Biobank. **Table S2.** Component definitions used in the UK Biobank study of the healthy lifestyle score. **Table S3.** Diet component definitions used in the UK Biobank study. **Table S4.** Healthy sleep pattern definitions used in the UK Biobank study. **Figure S1.** Flowchart for the Selection of the Analyzed Study Sample From the UK Biobank Study. **Table S5.** Hazard Ratios Adjusted with Different Models for T2DM Events According to Healthy Lifestyle Score. **Table S6.** Hazard Ratios for T2DM Events stratified by individual healthy lifestyle factors. **Table S7.** Multivariable adjusted hazard ratios for T2DM events according to healthy sleep score. **Table S8.** Hazard ratios for T2DM according to healthy lifestyle score among participants with dysglycaemia and hypertension. **Table S9.** Hazard ratios for T2DM excluding the first 2 years incidence cases according to healthy lifestyle score. **Table S10.** Hazard ratios for T2DM excluding BMI< 18.5kg/m^2^ incidence cases according to healthy lifestyle score. **Table S11.** Hazard ratios for T2DM excluding sleep apnea individuals at baseline. **Table S12.** Multivariable-adjusted PARs% (95% CIs) for incident T2DM by the specific combination of low-risk lifestyle factors among 430,971 participants.

## Data Availability

Data are available in a public, open access repository. This research has been conducted using the UK Biobank Resource under Application Number 44430. The UK Biobank data are available on application to the UK Biobank (www.ukbiobank.ac.uk/).

## Abbreviations

T2DM: Type 2 diabetes mellitus
PAR: Population attributable risk
FPG: Fasting plasma glucose
HbA1c: Haemoglobin A1c
BMI: Body mass index
WC: Waist circumference
ADA: American Diabetes Association
NHS: National Health Service
HES: Hospital Episode Statistics
SMR: Scottish Morbidity Record
PEDW: Patient Episode Database for Wales
ICD: International Classification of Diseases
HRs: Hazard ratios
CIs: Confidence intervals
NHS: Nurses’ Health Study
HPFS: Health Professionals Follow-up Study

## References

[1] Zheng Y, Ley SH, Hu FB (2018). Global aetiology and epidemiology of type 2 diabetes mellitus and its complications. Nat Rev Endocrinol.

[2] Conen D, Ridker PM, Mora S, Buring JE, Glynn RJ (2007). Blood pressure and risk of developing type 2 diabetes mellitus: the Women's Health Study. Eur Heart J.

[3] Wei GS, Coady SA, Goff DC, Brancati FL, Levy D, Selvin E (2011). Blood pressure and the risk of developing diabetes in african americans and whites: ARIC, CARDIA, and the framingham heart study. Diabetes Care.

[4] Emdin CA, Anderson SG, Woodward M, Rahimi K (2015). Usual blood pressure and risk of new-onset diabetes: evidence from 4.1 million adults and a meta-analysis of prospective studies. J Am Coll Cardiol.

[5] Ley SH, Hamdy O, Mohan V, Hu FB (2014). Prevention and management of type 2 diabetes: dietary components and nutritional strategies. Lancet.

[6] Guardado-Mendoza R, Salazar-López SS, Álvarez-Canales M, Farfán-Vázquez D, Martínez-López YE, Jiménez-Ceja LM (2020). The combination of linagliptin, metformin and lifestyle modification to prevent type 2 diabetes (PRELLIM). A randomized clinical trial. Metabolism.

[7] Li G, Zhang P, Wang J, An Y, Gong Q, Gregg EW (2014). Cardiovascular mortality, all-cause mortality, and diabetes incidence after lifestyle intervention for people with impaired glucose tolerance in the Da Qing Diabetes Prevention Study: a 23-year follow-up study. Lancet Diabetes Endocrinol.

[8] Jackson CL, Redline S, Kawachi I, Hu FB (2013). Association between sleep duration and diabetes in black and white adults. Diabetes Care.

[9] Cappuccio FP, D'Elia L, Strazzullo P, Miller MA (2010). Quantity and quality of sleep and incidence of type 2 diabetes: a systematic review and meta-analysis. Diabetes Care.

[10] Cedernaes J, Schiöth HB, Benedict C (2015). Determinants of shortened, disrupted, and mistimed sleep and associated metabolic health consequences in healthy humans. Diabetes.

[11] Wang M, Zhou T, Li X, Ma H, Liang Z, Fonseca VA (2020). Baseline vitamin D status, sleep patterns, and the risk of incident type 2 diabetes in data from the UK Biobank Study. Diabetes Care.

[12] Fan M, Sun D, Zhou T, Heianza Y, Lv J, Li L (2020). Sleep patterns, genetic susceptibility, and incident cardiovascular disease: a prospective study of 385 292 UK biobank participants. Eur Heart J.

[13] Roenneberg T, Wirz-Justice A, Merrow M (2003). Life between clocks: daily temporal patterns of human chronotypes. J Biol Rhythms.

[14] Baron KG, Reid KJ, Kern AS, Zee PC (2011). Role of sleep timing in caloric intake and BMI. Obesity (Silver Spring).

[15] Hakulinen C, Pulkki-Råback L, Virtanen M, Jokela M, Kivimäki M, Elovainio M (2018). Social isolation and loneliness as risk factors for myocardial infarction, stroke and mortality: UK Biobank cohort study of 479 054 men and women. Heart.

[16] Biobank. U. UK Biobank: protocol for a large-scale prospective epidemiological resource. http://www.ukbiobankacuk/wp-content/uploads/2011/11/UK-Biobank-Protocol.pdf. Accessed 15 Dec 2015.

[17] Biobank. U. UK Biobank ethics and governance framework. https://www.ukbiobankacuk/wp-content/uploads/2011/05/EGF20082.pdf. Accessed 15 Dec 2015.

[18] Sudlow C, Gallacher J, Allen N, Beral V, Burton P, Danesh J (2015). UK biobank: an open access resource for identifying the causes of a wide range of complex diseases of middle and old age. PLoS Med.

[19] Palmer LJ (2007). UK Biobank: bank on it. Lancet.

[20] Connelly K, Gandhi S, Horton E (2018). Lifestyle and CV risk in patients with diabetes: time to get "Back to Basics". J Am Coll Cardiol.

[21] Management L (2018). Standards of medical care in diabetes-2018. Diabetes Care.

[22] Dhana K, Nano J, Ligthart S, Peeters A, Hofman A, Nusselder W (2016). Obesity and life expectancy with and without diabetes in adults aged 55 years and older in the netherlands: a prospective cohort study. PLoS Med.

[23] Knott C, Bell S, Britton A (2015). Alcohol consumption and the risk of type 2 diabetes: a systematic review and dose-response meta-analysis of more than 1.9 million individuals from 38 observational studies. Diabetes Care.

[24] Lloyd-Jones DM, Hong Y, Labarthe D, Mozaffarian D, Appel LJ, Van Horn L (2010). Defining and setting national goals for cardiovascular health promotion and disease reduction: the American Heart Association's strategic Impact Goal through 2020 and beyond. Circulation.

[25] Mozaffarian D (2016). Dietary and policy priorities for cardiovascular disease, diabetes, and obesity: a comprehensive review. Circulation.

[26] Jarman B, Townsend P, Carstairs V (1991). Deprivation indices. BMJ.

[27] Jung JY, Oh CM, Ryoo JH, Choi JM, Choi YJ, Ham WT (2018). The influence of prehypertension, hypertension, and glycated hemoglobin on the development of type 2 diabetes mellitus in prediabetes: the Korean Genome and Epidemiology Study (KoGES). Endocrine.

[28] Li Y, Ley SH, Tobias DK, Chiuve SE, VanderWeele TJ, Rich-Edwards JW (2015). Birth weight and later life adherence to unhealthy lifestyles in predicting type 2 diabetes: prospective cohort study. BMJ.

[29] Hu FB, Manson JE, Stampfer MJ, Colditz G, Liu S, Solomon CG (2001). Diet, lifestyle, and the risk of type 2 diabetes mellitus in women. N Engl J Med.

[30] Lai YJ, Lin CL, Lin MC, Lee ST, Sung FC, Chang YJ (2013). Population-based cohort study on the increase in the risk for type 2 diabetes mellitus development from nonapnea sleep disorders. Sleep Med.

[31] Shan Z, Ma H, Xie M, Yan P, Guo Y, Bao W (2015). Sleep duration and risk of type 2 diabetes: a meta-analysis of prospective studies. Diabetes Care.

[32] Program TDP (1999). Design and methods for a clinical trial in the prevention of type 2 diabetes. Diabetes Care.

[33] McMurray JJ, Holman RR, Haffner SM, Bethel MA, Holzhauer B, Group NS (2010). Effect of valsartan on the incidence of diabetes and cardiovascular events. N Engl J Med.

[34] Gress TW, Nieto FJ, Shahar E, Wofford MR, Brancati FL (2000). Hypertension and antihypertensive therapy as risk factors for type 2 diabetes mellitus. Atherosclerosis Risk in Communities Study. N Engl J Med.

[35] Mozaffarian D, Kamineni A, Carnethon M, Djoussé L, Mukamal KJ, Siscovick D (2009). Lifestyle risk factors and new-onset diabetes mellitus in older adults: the cardiovascular health study. Arch Intern Med.

[36] Lv J, Yu C, Guo Y, Bian Z, Yang L, Chen Y (2017). Adherence to a healthy lifestyle and the risk of type 2 diabetes in Chinese adults. Int J Epidemiol.

[37] Steinbrecher A, Morimoto Y, Heak S, Ollberding NJ, Geller KS, Grandinetti A (2011). The preventable proportion of type 2 diabetes by ethnicity: the multiethnic cohort. Ann Epidemiol.

[38] St-Onge MP, McReynolds A, Trivedi ZB, Roberts AL, Sy M, Hirsch J (2012). Sleep restriction leads to increased activation of brain regions sensitive to food stimuli. Am J Clin Nutr.

[39] Schmid SM, Hallschmid M, Schultes B (2015). The metabolic burden of sleep loss. Lancet Diabetes Endocrinol.

[40] Buxton OM, Pavlova M, Reid EW, Wang W, Simonson DC, Adler GK (2010). Sleep restriction for 1 week reduces insulin sensitivity in healthy men. Diabetes.

[41] Sung KC, Lim S, Rosenson RS (2011). Hyperinsulinemia and homeostasis model assessment of insulin resistance as predictors of hypertension: a 5-year follow-up study of Korean sample. Am J Hypertens.

[42] Park SK, Jung JY, Choi WJ, Kim YH, Kim HS, Ham WT (2014). Elevated fasting serum insulin level predicts future development of hypertension. Int J Cardiol.

[43] Abdul-Ghani MA, Jayyousi A, DeFronzo RA, Asaad N, Al-Suwaidi J (2019). Insulin resistance the link between T2DM and CVD: basic mechanisms and clinical implications. Curr Vasc Pharmacol.

[44] Arcaro G, Cretti A, Balzano S, Lechi A, Muggeo M, Bonora E (2002). Insulin causes endothelial dysfunction in humans: sites and mechanisms. Circulation.

[45] Meigs JB, Hu FB, Rifai N, Manson JE (2004). Biomarkers of endothelial dysfunction and risk of type 2 diabetes mellitus. JAMA.

[46] Allen N, Sudlow C, Downey P, Peakman T, Danesh J, Elliott P (2012). UK Biobank: current status and what it means for epidemiology. Health Policy Technol.

[47] Batty GD, Gale CR, Kivimaki M, Deary IJ, Bell S (2020). Comparison of risk factor associations in UK Biobank against representative, general population based studies with conventional response rates: prospective cohort study and individual participant meta-analysis. BMJ.

[48] Manolio TA, Collins R (2010). Enhancing the feasibility of large cohort studies. JAMA.

