# Supplementary material for: Association of healthy lifestyle including a healthy sleep pattern with incident type 2 diabetes mellitus among individuals with hypertension
Source: Cardiovasc Diabetol. 2021 Dec 18;20:239. doi: 10.1186/s12933-021-01434-z (PMC8684653; doi:10.1186/s12933-021-01434-z)
Supplement: Supplementary file 1 — Additional file 1: Table S1. Disease codes for Metabolic diseases of Type 2 Diabetes, Hypertension, Cardiovascular disease in UK Biobank. Table S2. Component definitions used in the UK Biobank study of the healthy lifestyle score. Table S3. Diet component definitions used in the UK Biobank study. Table S4. Healthy sleep pattern definitions used in the UK Biobank study. Figure S1. Flowchart for the Selection of the Analyzed Study Sample From the UK Biobank Study. Table S5. Hazard Ratios Adjusted with Different Models for T2DM Events According to Healthy Lifestyle Score. Table S6. Hazard Ratios for T2DM Events stratified by individual healthy lifestyle factors. Table S7. Multivariable adjusted hazard ratios for T2DM events according to healthy sleep score. Table S8. Hazard ratios for T2DM according to healthy lifestyle score among participants with dysglycaemia and hypertension. Table S9. Hazard ratios for T2DM excluding the first 2 years incidence cases according to healthy lifestyle score. Table S10. Hazard ratios for T2DM excluding BMI< 18.5kg/m2 incidence cases according to healthy lifestyle score. Table S11. Hazard ratios for T2DM excluding sleep apnea individuals at baseline. Table S12. Multivariable-adjusted PARs% (95% CIs) for incident T2DM by the specific combination of low-risk lifestyle factors among 430,971 participants. [file 12933_2021_1434_MOESM1_ESM.docx]

**Additional file**

Table S1. Disease codes for Metabolic diseases of Type 2 Diabetes, Hypertension, Cardiovascular disease in UK Biobank

Table S2. Component definitions used in the UK Biobank study of the healthy lifestyle score

Table S3. Diet component definitions used in the UK Biobank study

Table S4. Healthy sleep pattern definitions used in the UK Biobank study

Figure S1. Flowchart for the Selection of the Analyzed Study Sample From the UK Biobank Study

Table S5. Hazard Ratios Adjusted with Different Models for T2DM Events According to Healthy Lifestyle Score

Table S6. Hazard Ratios for T2DM Events stratified by individual healthy lifestyle factors

Table S7. Multivariable adjusted hazard ratios for T2DM events according to healthy sleep score

Table S8. Hazard ratios for T2DM according to healthy lifestyle score among participants with dysglycaemia and hypertension

Table S9. Hazard ratios for T2DM excluding the first 2 years incidence cases according to healthy lifestyle score

Table S10. Hazard ratios for T2DM excluding BMI< 18.5kg/m^2^ incidence cases according to healthy lifestyle score

Table Ss11. Hazard ratios for T2DM excluding sleep apnea individuals at baseline

Table S12. Multivariable-adjusted PARs% (95% CIs) for incident T2DM by the specific combination of low-risk lifestyle factors among 430,971 participants

Table S1. Disease codes for Metabolic diseases of Type 2 Diabetes, Hypertension, Cardiovascular disease in UK Biobank

| Disease | Diagnoses of ICD-10-CM  (Variable ID:41202 and 41204) | Diagnoses of ICD-9-CM  (Variable ID:41203 and 41205) | Non-cancer illness  (Variable ID:20002) |
| --- | --- | --- | --- |
| Diabetes | E10-E14 | 250 | 1220 |
| Type 2 diabetes | E11 | 25000, 25010，25020，25090 | 1223 |
| Hypertension  Primary hypertension  Secondary hypertension | I10  I15 | 401  405 | 1065  1072 |
| Cardiovascular disease | I00-I99  I20-I21, I25, I48, I50, I63-I64，I672，I70 | 390-459  410-413, 4140, 4273, 428,  434-435，4370，440， | 1066, 1074-1076, 1081-1083, 1583 |

Table S2. Component definitions used in the UK Biobank study of the healthy lifestyle score

| Lifestyle factors | Classification of healthy lifestyle score | | | UK Biobank field code |
| --- | --- | --- | --- | --- |
|  | Poor | Intermediate | Ideal |  |
| BMI | ≥ 30.0 kg/m^2^ | 25.0-29.9 kg/m^2^ | < 25.0 kg/m^2^ | 21001 |
| Cigarette smoking | Current | Previous | never smoker | 20116 |
| Alcohol consumption | Women＞14g/day;  Men＞28g/day | never | 0<women≤14g/day; 0<men≤28g/day | 20117,1558,1568,1578,1588  1598,1608,4407,4418,4429,4440,4451 |
| Physical activity | No report of moderate or vigorous physical activity | 0 < mins of moderate physical activity < 150; or  0 < mins of moderate < 75; or  0 < mins of combined moderate and vigorous physical activity < 150 | ≥150 mins of moderate physical activity; or  ≥75 mins of vigorous physical activity; or  ≥150 mins of combined moderate and vigorous physical activity | 884, 894, 904, 914 |
| Healthy diet score^a^ | 0-1 components | 2-3 components | 4-5 components | 1309,1319,1289,1299,1329,1339,1349,1369,1379,1389,1438,1448,1458,1468 |
| Sleep pattern score | 0-1 points | 2-3 points | 4-5 points | 1160,1180,1200,1210,1220 |
| Points for lifestyle score per Metric | 0 | 0 | 1 |  |

Abbreviations: BMI=indicates body mass index. ^a^Adapted for the study;

Table S3. Diet component definitions used in the UK Biobank study

| Diet component | Intake goal | Field IDs | Amount per serving |
| --- | --- | --- | --- |
| Fruit | ≥3 servings/day | 1309 (pieces fresh fruit/day) 1319 (pieces dried fruit/day) | 1309–1 piece 1319–5 pieces |
|  |  |  |  |
| Vegetable | ≥3 servings/day | 1289 (tablespoons cooked vegetables/day) 1299 (salad/raw vegetables/day) | 3 heaped tablespoons |
| (Shell)fish | ≥2 servings/week | 1329 (oily fish/week) 1339 (non-oily fish/week) | Once/week |
|  |  |  |  |
| Processed meats | ≤1 serving/week | 1349 (processed meat/week or daily) 3680 (age when last ate meat) | 1349–1 piece/day  3680–0 pieces/day if indicated having never eaten meat |
| Unprocessed meats | ≤2 serving/week | 1369 (beef/week or day) 1379 (lamb or mutton/week or day) 1389 (pork/week or day) 3680 (age when last ate meat) | 1359-1389–once/week  3680–0 pieces/day if indicated having never eaten meat |

Field IDs and serving sizes used per diet component in UK Biobank with available data from the general baseline questionnaire. If participants achieved the intake goal, they were considered adequate intake of the diet component.

Table S4. Healthy sleep pattern definitions used in the UK Biobank study

| Sleep behaviours | High-risk sleep factors^a^ | Low-risk sleep factors | Field IDs |
| --- | --- | --- | --- |
| Chronotype preference | more an “evening” than “morning” person  definitely an “evening” person | definitely a “morning” person  more a “morning” than “evening” person | 1180 |
| Sleep duration | short (<7h/day)  long (>=9h/day) | normal (7–8h/day) | 1160 |
| Insomnia symptoms | Sometimes  usually | Never/rarely | 1200 |
| Information on snoring | yes | no | 1210 |
| daytime sleepiness | Often  all of the time | never/rarely  sometimes | 1220 |

^a^For each sleep factor, and the participant received a score of 1 if he or she was classified as low risk for that factor or 0 if at high risk for that factor.

Figure S1. Flowchart for the selection of the analyzed study sample from the UK Biobank study


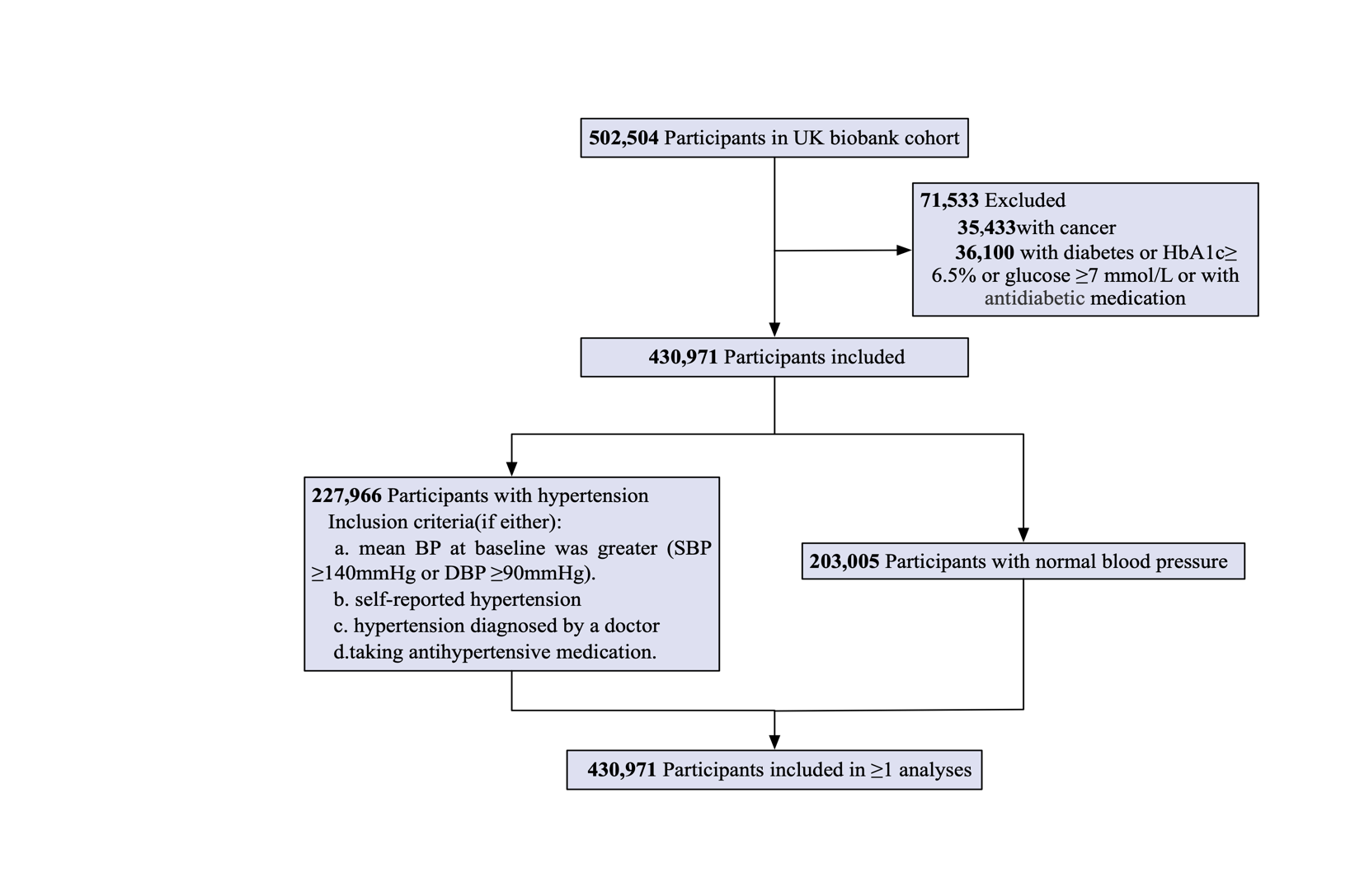


Table S5. Hazard Ratios Adjusted with Different Models for T2DM Events According to Healthy Lifestyle Score

| Healthy lifestyle score | No. of cases^a^ | Person-Years | Cases/PYs (1,000) | HR (95% CI)^e^ | | | | PAR% (95%CI) |
| --- | --- | --- | --- | --- | --- | --- | --- | --- |
|  |  |  |  | Model 1^b^ | Model 2^c^ | Model 3^d^ | Model 4^e^ |  |
| Hypertension |  |  |  |  |  |  |  |  |
| 0 | 393 | 68699.97 | 5.72 | 1 (reference) | 1 (reference) | 1 (reference) | 1 (reference) | NA |
| 1 | 1,127 | 282608.1 | 3.99 | 0.69 (0.62-0.78) | 0.71 (0.63-0.80) | 0.77 (0.67-0.87) | 0.86 (0.75-0.97) | NA |
| 2 | 1,283 | 458167.7 | 2.80 | 0.49 (0.43-0.54) | 0.52 (0.47-0.59) | 0.59 (0.52-0.67) | 0.75 (0.66-0.85) | NA |
| 3 | 792 | 420736.8 | 1.88 | 0.33 (0.29-0.37) | 0.36 (0.32-0.41) | 0.42 (0.37-0.49) | 0.63 (0.55-0.72) | 24.66 (20.06-29.00) |
| 4 | 273 | 231402.9 | 1.18 | 0.20 (0.18-0.24) | 0.24 (0.21-0.28) | 0.29 (0.25-0.35) | 0.53 (0.44-0.63) | 49.80 (44.09-54.92) |
| ≥5 | 49 | 79122.95 | 0.62 | 0.11 (0.08-0.14) | 0.14 (0.10-0.19) | 0.17 (0.13-0.24) | 0.41 (0.30-0.57) | 71.37 (62.50-78.14) |
| Per unit increase in healthy lifestyle score | NA | NA | NA | 0.67 (0.65-0.69) | 0.70 (0.68-0.72) | 0.73 (0.71-0.76) | 0.85 (0.83-0.88) | NA |
| p for interaction^f^ |  |  |  | 0.0002 | 0.0011 | 0.0001 | 0.0013 |  |
| Normotension |  |  |  |  |  |  |  |  |
| 0 | 87 | 38669.89 | 2.25 | 1 (reference) | 1 (reference) | 1 (reference) | 1 (reference) | NA |
| 1 | 288 | 175931.1 | 1.64 | 0.73 (0.57-0.92) | 0.76 (0.59-0.96) | 0.83 (0.64-1.08) | 0.99 (0.76-1.29) | NA |
| 2 | 355 | 352766.1 | 1.01 | 0.45 (0.35-0.56) | 0.49 (0.39-0.62) | 0.56 (0.43-0.72) | 0.78 (0.60-1.01) | NA |
| 3 | 260 | 411834.6 | 0.63 | 0.28 (0.22-0.36) | 0.34 (0.26-0.43) | 0.39 (0.30-0.51) | 0.66 (0.51-0.87) | 17.53 (8.32-25.82) |
| 4 | 89 | 302752.5 | 0.29 | 0.13 (0.10-0.17) | 0.17 (0.13-0.23) | 0.20 (0.14-0.27) | 0.42 (0.30-0.59) | 58.75 (50.71-65.48) |
| ≥5 | 27 | 135723.8 | 0.20 | 0.09 (0.06-0.14) | 0.13 (0.08-0.19) | 0.16 (0.10-0.25) | 0.44 (0.27-0.70) | 69.43 (56.49-78.53) |
| Per unit increase in healthy lifestyle score | NA | NA | NA | 0.60 (0.58-0.63) | 0.65 (0.62-0.68) | 0.67 (0.64-0.71) | 0.81 (0.77-0.86) | NA |
| All Participants |  |  |  |  |  |  |  |  |
| 0 | 480 | 107369.9 | 4.47 | 1 (reference) | 1 (reference) | 1 (reference) | 1 (reference) | NA |
| 1 | 1,415 | 458539.2 | 3.09 | 0.69 (0.62-0.76) | 0.71 (0.64-0.79) | 0.77 (0.69-0.87) | 0.88 (0.79-0.99) | NA |
| 2 | 1,638 | 810933.8 | 2.02 | 0.45 (0.41-0.50) | 0.50 (0.45-0.55) | 0.57 (0.51-0.64) | 0.75 (0.67-0.84) | NA |
| 3 | 1,052 | 832571.4 | 1.26 | 0.28 (0.25-0.31) | 0.34 (0.30-0.38) | 0.40 (0.35-0.45) | 0.63 (0.56-0.71) | 23.85 (19.79-27.70) |
| 4 | 362 | 534155.4 | 0.68 | 0.15 (0.13-0.17) | 0.20 (0.17-0.23) | 0.24 (0.21-0.28) | 0.48 (0.41-0.56) | 55.06 (50.75-58.99) |
| ≥5 | 76 | 214846.8 | 0.35 | 0.08 (0.06-0.10) | 0.12 (0.09-0.15) | 0.15 (0.12-0.20) | 0.39 (0.30-0.51) | 73.74 (67.47-78.80) |
| Per unit increase in healthy lifestyle score | NA | NA | NA | 0.62 (0.61-0.64) | 0.67 (0.66-0.69) | 0.70 (0.68-0.72) | 0.84 (0.81-0.86) | NA |

Abbreviations: HR=Hazard Ratio; CI=Confidence Interval; NA=Not Applicable; PAR%= Population attributable risk%

^a^Cases are incident cases of diabetes during the study.

^b^Model1 is unadjusted. Low-risk lifestyle factors: BMI of ≤ 24.9 kg/m2; moderate alcohol consumption (0 to 15 g/day for women and 0 to 28 g/day for men). Nonsmoking; moderate to vigorous physical activity ($150 min/week), high-quality diet (top two-fifths of healthy diet score), and healthy sleep pattern (top two-fifths of healthy diet score).

^c^Model2 is adjusted for age, sex, education, socioeconomic status, parental history of T2DM or hypertension.

^d^Model3 is adjusted for age, sex, education, socioeconomic status, parental history of T2DM or hypertension, cholesterol-lowering drugs, hypercholesterolemia, glucose level (normal glucose regulation, prediabetes).

^e^Model3 is adjusted for age, sex, education, socioeconomic status, parental history of T2DM or hypertension, cholesterol-lowering drugs, hypercholesterolemia, glucose level (normal glucose regulation, prediabetes), waist circumference.

^f^All P_trend_ ＜0.001.

^g^ P for interaction calculated using multiplicative interaction terms and applying the likelihood ratio test: interaction between normotension and hypertension.

Table S6. Hazard Ratios for T2DM Events stratified by individual healthy lifestyle factors^a^

|  | overall | | |  | Normotension | | |  | Hypertension | | |
| --- | --- | --- | --- | --- | --- | --- | --- | --- | --- | --- | --- |
| Category | No. of Cases^b^ | Cases/PY^c^ | HR (95% CI)^d^ |  | No. of Cases | Cases/PY | HR (95% CI) |  | No. of Cases | Cases/PY | HR (95% CI) |
| Body mass index |  |  |  |  |  |  |  |  |  |  |  |
| ≥30.0 kg/m^2^ | 3,827 | 4.67 | 1(reference) |  | 649 | 2.57 | 1(reference) |  | 3,178 | 5.61 | 1(reference) |
| 25.0-29.9 kg/m^2^ | 2,410 | 1.51 | 0.36(0.34-0.38) |  | 630 | 0.89 | 0.40(0.35-0.46) |  | 1,780 | 2.00 | 0.38(0.35-0.40) |
| ＜25.0 kg/m2 | 624 | 0.49 | 0.15(0.13-0.16) |  | 220 | 0.28 | 0.15(0.12-0.18) |  | 404 | 0.83 | 0.18(0.16-0.21) |
| p_trend_ Value | ＜0.0001 | | |  | ＜0.0001 | | |  | ＜0.0001 | | |
| p for interaction^e^ | 0.4315 | | | | | | | | | | |
| Alcohol consumption |  |  |  |  |  |  |  |  |  |  |  |
| Never | 2,538 | 2.83 | 1(reference) |  | 594 | 1.35 | 1(reference) |  | 1,944 | 4.24 | 1(reference) |
| Moderate | 2,842 | 1.57 | 0.65(0.60-0.69) |  | 660 | 0.74 | 0.65(0.56-0.74) |  | 2,182 | 2.36 | 0.65(0.60-0.70) |
| Excessive | 1,535 | 1.54 | 0.60(0.55-0.65) |  | 259 | 0.61 | 0.55(0.46-0.66) |  | 1,276 | 2.25 | 0.59(0.54-0.64) |
| p_trend_ Value | ＜0.0001 | | |  | ＜0.0001 | | |  | ＜0.0001 | | |
| p for interaction^d^ | 0.2787 | | | | | | | | | | |
| Smoking status |  |  |  |  |  |  |  |  |  |  |  |
| Current smoker | 1,106 | 2.85 | 1(reference) |  | 314 | 1.56 | 1(reference) |  | 792 | 4.23 | 1(reference) |
| Former smoker | 2,781 | 2.24 | 0.69(0.64-0.76) |  | 531 | 0.99 | 0.68(0.57-0.82) |  | 2,250 | 3.18 | 0.68(0.62-0.75) |
| Never smoker | 3,002 | 1.45 | 0.57(0.52-0.62) |  | 663 | 0.65 | 0.59(0.49-0.70) |  | 2,339 | 2.23 | 0.54(0.49-0.60) |
| p_trend_ Value | ＜0.0001 | | |  | ＜0.0001 | | |  | ＜0.0001 | | |
| p for interaction | 0.6167 | | | | | | | | | | |
| Physical activity |  |  |  |  |  |  |  |  |  |  |  |
| Poor | 1,313 | 3.00 | 1(reference) |  | 260 | 1.33 | 1(reference) |  | 1053 | 4.35 | 1(reference) |
| Intermediate | 1,234 | 2.12 | 0.81(0.74-0.89) |  | 263 | 0.99 | 0.83(0.67-1.02) |  | 971 | 3.06 | 0.81(0.73-0.90) |
| Ideal | 4,015 | 1.53 | 0.70(0.65-0.76) |  | 909 | 0.71 | 0.73(0.62-0.86) |  | 3106 | 2.31 | 0.70(0.64-0.76) |
| p_trend_ Value | ＜0.0001 | | |  | ＜0.0001 | | |  | ＜0.0001 | | |
| p for interaction | 0.6682 | | | | | | | | | | |
| Healthy diet score |  |  |  |  |  |  |  |  |  |  |  |
| 0-1 components | 2,349 | 2.16 | 1(reference) |  | 489 | 0.98 | 1(reference) |  | 1,860 | 3.15 | 1(reference) |
| 2-3 components | 3,726 | 1.76 | 0.96(0.90-1.02) |  | 851 | 0.84 | 1.03(0.90-1.17) |  | 2,875 | 2.62 | 0.95(0.88-1.01) |
| 4-5 components | 690 | 1.47 | 0.89(0.81-0.99) |  | 133 | 0.58 | 0.74(0.59-0.94) |  | 557 | 2.33 | 0.93(0.83-1.04) |
| p_trend_ Value | 0.030 | | |  | 0.089 | | |  | 0.102 | | |
| p for interaction | 0.7408 | | | | | | | | | | |
| Sleep score |  |  |  |  |  |  |  |  |  |  |  |
| 0-1 points | 537 | 4.29 | 1(reference) |  | 123 | 2.42 | 1(reference) |  | 414 | 5.57 | 1(reference) |
| 2-3 points | 3,405 | 1.94 | 0.59(0.54-0.65) |  | 728 | 0.91 | 0.50(0.41-0.62) |  | 2,677 | 2.81 | 0.63(0.56-0.70) |
| 4-5 points | 1,427 | 1.23 | 0.48(0.43-0.54) |  | 340 | 0.57 | 0.41(0.33-0.51) |  | 1,087 | 1.93 | 0.52(0.46-0.59) |
| p_trend_ Value | ＜0.0001 | | |  | ＜0.0001 | | |  | ＜0.0001 | | |
| p for interaction | 0.1988 | | | | | | | | | | |

Abbreviations: No=Number; T2DM=Type 2 Diabetes Mellitus; HR= Hazard Ratio; CI= Confidence Interval; PY=person year.

^a^203,005participants with normotension and 227,966 with hypertension were included in the analysis.

^b^Cases are incident cases of Type 2 diabetes mellitus during the study.

^c^Adjusted for age, sex, education, socioeconomic status, parental history of T2DM or hypertension.

^d^Incidence density per 1000 person-years.

^e^P for interaction calculated using multiplicative interaction terms and applying the likelihood ratio test: interaction between normotension and hypertension.

Table S7. Multivariable adjusted hazard ratios for T2DM events according to healthy sleep score

| Healthy sleep score | No. of cases^a^ | Person-Years | Cases/PYs (1,000) | HR (95% CI) | |
| --- | --- | --- | --- | --- | --- |
|  |  |  |  | Model 1^b^ | Model 2^c^ |
| Hypertension |  |  |  |  |  |
| 0 | 34 | 68699.97 | 5.72 | 1 (reference) | 1 (reference) |
| 1 | 380 | 282608.1 | 3.99 | 0.62 (0.43-0.88) | 0.55 (0.38-0.80) |
| 2 | 1,149 | 458167.7 | 2.80 | 0.40 (0.28-0.57) | 0.39 (0.27-0.57) |
| 3 | 1,528 | 420736.8 | 1.88 | 0.30 (0.21-0.42) | 0.30 (0.21-0.43) |
| 4 | 916 | 231402.9 | 1.18 | 0.24 (0.17-0.34) | 0.25 (0.17-0.36) |
| 5 | 171 | 79122.95 | 0.62 | 0.21 (0.15-0.31) | 0.22 (0.15-0.32) |
| Per unit increase in healthy sleep score | NA | NA | NA | 0.76 (0.73-0.78) | 0.78 (0.75-0.81) |
| Normotension |  |  |  |  |  |
| 0 | 12 | 38669.89 | 2.25 | 1 (reference) | 1 (reference) |
| 1 | 111 | 175931.1 | 1.64 | 0.46 (0.25-0.83) | 0.54 (0.28-1.03) |
| 2 | 292 | 352766.1 | 1.01 | 0.24 (0.13-0.43) | 0.29 (0.15-0.54) |
| 3 | 436 | 411834.6 | 0.63 | 0.18 (0.10-0.32) | 0.22 (0.12-0.42) |
| 4 | 287 | 302752.5 | 0.29 | 0.15 (0.08-0.26) | 0.19 (0.10-0.36) |
| 5 | 53 | 135723.8 | 0.20 | 0.11 (0.06-0.21) | 0.13 (0.07-0.26) |
| Per unit increase in healthy sleep score | NA | NA | NA | 0.72 (0.68-0.77) | 0.74 (0.70-0.79) |
| p for interaction^d^ |  |  |  | 0.0823 | 0.0334 |
| All Participants |  |  |  |  |  |
| 0 | 46 | 107369.9 | 4.47 | 1 (reference) | 1 (reference) |
| 1 | 491 | 458539.2 | 3.09 | 0.57 (0.42-0.77) | 0.55 (0.40-0.76) |
| 2 | 1,441 | 810933.8 | 2.02 | 0.35 (0.26-0.47) | 0.36 (0.27-0.50) |
| 3 | 1,964 | 832571.4 | 1.26 | 0.25 (0.19-0.34) | 0.27 (0.20-0.37) |
| 4 | 1,203 | 534155.4 | 0.68 | 0.20 (0.15-0.27) | 0.23 (0.16-0.31) |
| 5 | 224 | 214846.8 | 0.35 | 0.17 (0.12-0.23) | 0.18 (0.13-0.26) |
| Per unit increase in healthy sleep score | NA | NA | NA | 0.74 (0.72-0.76) | 0.76 (0.74-0.78) |

Abbreviations: HR=Hazard Ratio; CI=Confidence Interval; NA=Not Applicable.

^a^Cases are incident cases of diabetes during the study.

^b^Model1 is adjusted for age, sex, education, socioeconomic status, parental history of T2DM or hypertension.

^c^Model2 is adjusted for age, sex, education, socioeconomic status, parental history of T2DM or hypertension, cholesterol-lowering drugs, hypercholesterolemia, glucose level (normal glucose regulation, prediabetes). All Ptrend ＜0.001.

^d^P for interaction calculated using multiplicative interaction terms and the likelihood ratio test: interaction between normotension and hypertension.

Table S8. Hazard ratios for T2DM according to healthy lifestyle score among participants with dysglycaemia and hypertension

| healthy lifestyle score | HR (95% CI)^a^ | | | | | | |
| --- | --- | --- | --- | --- | --- | --- | --- |
|  | Normotension + normoglycemia |  | Hypertension only |  | IFG only |  | Hypertension+ IFG |
| 0 | 1(reference) |  | 1(reference) |  | 1(reference) |  | 1(reference) |
| 1 | 0.75(0.57-1.00) |  | 0.70(0.61-0.80) |  | 0.99(0.61-1.60) |  | 0.81(0.64-1.01) |
| 2 | 0.51(0.38-0.67) |  | 0.50(0.43-0.57) |  | 0.61(0.38-0.98) |  | 0.71(0.57-0.89) |
| 3 | 0.37(0.28-0.50) |  | 0.36(0.31-0.42) |  | 0.35(0.21-0.59) |  | 0.49(0.39-0.63) |
| 4 | 0.16(0.11-0.22) |  | 0.22(0.18-0.27) |  | 0.36(0.21-0.68) |  | 0.47(0.35-0.63) |
| ≥5 | 0.15(0.09-0.24) |  | 0.15(0.10-0.20) |  | 0.09(0.02-0.37) |  | 0.22(0.11-0.42) |
| Each 1-number increment | 0.66(0.62-0.70) |  | 0.69(0.67-0.72) |  | 0.69(0.61-0.77) |  | 0.80(0.76-0.84) |

Abbreviations: T2DM= Type 2 Diabetes Mellitus; IFG= Impaired Fasting Glucose

^a^Adjusted for age, sex, education, socioeconomic status quintile, parental history of T2DM or hypertension. Low-risk lifestyle factors: BMI of ≤ 24.9 kg/m2; moderate alcohol consumption (0 to 15 g/day for women and 0 to 28 g/day for men). Nonsmoking; moderate to vigorous physical activity ($150 min/week), high-quality diet (top two-fifths of healthy diet score), and healthy sleep pattern (top two-fifths of healthy diet score).

Table S9. Hazard ratios for T2DM excluding the first 2 years incidence cases according to healthy lifestyle score

| Healthy lifestyle score | No. of cases | Person-Years | Cases/PYs (1,000) | HR (95% CI)* |
| --- | --- | --- | --- | --- |
| Hypertension |  |  |  |  |
| 0 | 361 | 68658.5 | 5.26 | 1(reference) |
| 1 | 1,048 | 282507 | 3.71 | 0.72 (0.64-0.81) |
| 2 | 1,179 | 458047 | 2.57 | 0.52 (0.46-0.59) |
| 3 | 730 | 420661 | 1.74 | 0.37 (0.32-0.42) |
| 4 | 249 | 231374 | 1.08 | 0.24 (0.20-0.28) |
| ≥5 | 43 | 79114 | 0.54 | 0.13 (0.10-0.18) |
| Per unit increase in healthy lifestyle score | NA | NA | NA | 0.70 (0.68-0.72) |
| Normotension |  |  |  |  |
| 0 | 78 | 38657.3 | 2.02 | 1(reference) |
| 1 | 276 | 175918 | 1.57 | 0.81 (0.63-1.04) |
| 2 | 333 | 352738 | 0.94 | 0.52 (0.40-0.66) |
| 3 | 239 | 411805 | 0.58 | 0.35 (0.27-0.45) |
| 4 | 79 | 302740 | 0.26 | 0.17 (0.12-0.23) |
| ≥5 | 25 | 135723 | 0.18 | 0.13 (0.08-0.21) |
| Per unit increase in healthy lifestyle score | NA | NA | NA | 0.64 (0.61-0.68) |
| All Participants |  |  |  |  |
| 0 | 439 | 107316 | 4.09 | 1(reference) |
| 1 | 1,324 | 458425 | 2.89 | 0.73 (0.66-0.81) |
| 2 | 1,512 | 810785 | 1.86 | 0.50 (0.45-0.56) |
| 3 | 969 | 832466 | 1.16 | 0.34 (0.30-0.38) |
| 4 | 328 | 534114 | 0.61 | 0.20 (0.17-0.23) |
| ≥5 | 68 | 214837 | 0.32 | 0.11 (0.09-0.15) |
| Per unit increase in healthy lifestyle score | NA | NA | NA | 0.67 (0.65-0.68) |

Abbreviations: T2DM= Type 2 Diabetes Mellitus; No.=Number; HR= Hazard Ratio; CI= Confidence Interval; PY=person year.

*Adjusted for age, sex, education, socioeconomic status, parental history of T2DM or hypertension.Low-risk lifestyle factors: BMI of ≤ 24.9 kg/m2; moderate alcohol consumption (0 to 15 g/day for women and 0 to 28 g/day for men). Nonsmoking; moderate to vigorous physical activity ($150 min/week), high-quality diet (top two-fifths of healthy diet score), and healthy sleep pattern (top two-fifths of healthy diet score).

Table S10. Hazard ratios for T2DM excluding BMI< 18.5kg/m^2^ incidence cases according to healthy lifestyle score

| Healthy lifestyle score | No. of cases | Person-Years | Cases/PYs (1,000) | HR (95% CI)* |
| --- | --- | --- | --- | --- |
| Hypertension |  |  |  |  |
| 0 | 393 | 68699.97 | 5.72 | 1(reference) |
| 1 | 1,126 | 282292.3 | 3.99 | 0.71 (0.63-0.80) |
| 2 | 1,282 | 457348.7 | 2.80 | 0.52 (0.47-0.59) |
| 3 | 792 | 419627.2 | 1.89 | 0.37 (0.32-0.41) |
| 4 | 272 | 230196.2 | 1.18 | 0.24 (0.21-0.28) |
| ≥5 | 49 | 78539.94 | 0.62 | 0.14 (0.10-0.19) |
| Per unit increase in healthy lifestyle score | NA | NA | NA | 0.70 (0.68-0.72) |
| Normotension |  |  |  |  |
| 0 | 87 | 38669.89 | 2.25 | 1(reference) |
| 1 | 288 | 175371.6 | 1.64 | 0.76 (0.60-0.96) |
| 2 | 353 | 350867.3 | 1.01 | 0.49 (0.39-0.62) |
| 3 | 260 | 408498.8 | 0.64 | 0.34 (0.27-0.44) |
| 4 | 87 | 299395.5 | 0.29 | 0.17 (0.12-0.23) |
| ≥5 | 26 | 133487.8 | 0.19 | 0.12 (0.08-0.19) |
| Per unit increase in healthy lifestyle score | NA | NA | NA | 0.65 (0.62-0.68) |
| All Participants |  |  |  |  |
| 0 | 480 | 107369.9 | 4.47 | 1(reference) |
| 1 | 1,414 | 457663.9 | 3.09 | 0.71 (0.64-0.79) |
| 2 | 1,635 | 808216 | 2.02 | 0.50 (0.45-0.55) |
| 3 | 1,052 | 828126 | 1.27 | 0.34 (0.30-0.38) |
| 4 | 359 | 529591.7 | 0.68 | 0.20 (0.17-0.23) |
| ≥5 | 75 | 212027.8 | 0.35 | 0.12 (0.09-0.15) |
| Per unit increase in healthy lifestyle score | NA | NA | NA | 0.67 (0.66-0.69) |

Abbreviations: T2DM= Type 2 Diabetes Mellitus; No.=Number; HR= Hazard Ratio; CI= Confidence Interval; PY=person year.

*Adjusted for age, sex, education, socioeconomic status, parental history of T2DM or hypertension. Low-risk lifestyle factors: BMI of ≤ 24.9 kg/m2; moderate alcohol consumption (0 to 15 g/day for women and 0 to 28 g/day for men). Nonsmoking; moderate to vigorous physical activity ($150 min/week), high-quality diet (top two-fifths of healthy diet score), and healthy sleep pattern (top two-fifths of healthy diet score)

Table S11. Hazard ratios for T2DM excluding sleep apnea individuals at baseline

| Healthy lifestyle score | No. of cases | Person-Years | Cases/PYs (1,000) | HR (95% CI)* |
| --- | --- | --- | --- | --- |
| Hypertension |  |  |  |  |
| 0 | 386 | 68190.17 | 5.66 | 1(reference) |
| 1 | 1,109 | 281194.3 | 3.94 | 0.71 (0.63 -0.80) |
| 2 | 1,265 | 456495.9 | 2.77 | 0.52 (0.47 -0.59) |
| 3 | 777 | 419534.5 | 1.85 | 0.36 (0.32 -0.41) |
| 4 | 272 | 231110 | 1.18 | 0.25 (0.21 -0.29) |
| ≥5 | 48 | 79050.21 | 0.61 | 0.14 (0.10 -0.19) |
| Per unit increase in healthy lifestyle score | NA | NA | NA | 0.70 (0.68 -0.72) |
| Normotension |  |  |  |  |
| 0 | 85 | 38464.23 | 2.21 | 1(reference) |
| 1 | 283 | 175413.5 | 1.61 | 0.76 (0.59 -0.97) |
| 2 | 354 | 352059.1 | 1.01 | 0.50 (0.40 -0.64) |
| 3 | 259 | 411257.1 | 0.63 | 0.34 (0.27 -0.44) |
| 4 | 89 | 302521.9 | 0.29 | 0.17 (0.13 -0.23) |
| ≥5 | 27 | 135688.8 | 0.20 | 0.13 (0.08 -0.20) |
| Per unit increase in healthy lifestyle score | NA | NA | NA | 0.65 (0.62 -0.69) |
| All Participants |  |  |  |  |
| 0 | 471 | 106654.4 | 4.42 | 1(reference) |
| 1 | 1,392 | 456607.9 | 3.05 | 0.71 (0.64 -0.79) |
| 2 | 1,619 | 808555 | 2.00 | 0.50 (0.45 -0.56) |
| 3 | 1,036 | 830791.6 | 1.25 | 0.34 (0.30 -0.38) |
| 4 | 361 | 533631.9 | 0.68 | 0.20 (0.18 -0.23) |
| ≥5 | 75 | 214739 | 0.35 | 0.12 (0.09 -0.15) |
| Per unit increase in healthy lifestyle score | NA | NA | NA | 0.67 (0.66 -0.69) |

Abbreviations: T2DM= Type 2 Diabetes Mellitus; No.=Number; HR= Hazard Ratio; CI= Confidence Interval; PY=person year.

*Adjusted for age, sex, education, socioeconomic status, parental history of T2DM or hypertension. Low-risk lifestyle factors: BMI of ≤ 24.9 kg/m2; moderate alcohol consumption (0 to 15 g/day for women and 0 to 28 g/day for men). Nonsmoking; moderate to vigorous physical activity ($150 min/week), high-quality diet (top two-fifths of healthy diet score), and healthy sleep pattern (top two-fifths of healthy diet score)

Table S12. Multivariable-adjusted PARs% (95% CIs) for incident T2DM by a specific combination of low-risk lifestyle factors among 430,971 participants*

| Category | population attributable risk per cent (95% CIs) | | |
| --- | --- | --- | --- |
|  | hypertension | normotension | All participants |
| 3 factors in low-risk group^a^ | 75.43 (72.69-77.90) | 73.21 (69.38-76.56) | 77.20 (75.26-78.98) |
| 4 factors in low-risk group^b^ | 78.13 (75.69-80.34) | 75.59 (72.06-78.68) | 79.63 (77.89-81.23) |
| 5 factors in low-risk group^c^ | 80.43 (78.16-82.46) | 78.08 (74.73-80.98) | 81.96 (80.37-83.43) |
| 5-factors without the sleep pattern | 76.93 (73.56-79.87) | 80.06 (75.10-84.04) | 79.72 (77.31-81.87) |
| All 6 factors in the low-risk group | 81.14 (78.29-83.61) | 83.66 (79.45-87.00) | 83.51 (81.49-85.31) |

Abbreviations: PAR%=population attributable risk percent; CI=confidence interval;

*Multivariable model was adjusted for age, sex, education, socioeconomic status, parental history of T2DM or hypertension.

^a^Three lifestyle factors included BMI, healthy sleep pattern, and smoking. The model was additionally adjusted for physical activity, healthy diet and alcohol consumption.

^b^Four lifestyle factors included three above plus physical activity. The model was additionally adjusted for healthy diet and alcohol consumption.

^c^Five lifestyle factors included four above plus alcohol consumption. The model was additionally adjusted for a healthy diet.
